# Prevalence and Risk Factors for Alcohol Use Among Tunisian Nursing Students

**DOI:** 10.1192/j.eurpsy.2026.10904

**Published:** 2026-07-31

**Authors:** A. Sdiri, I. Betbout, D. Charfi, Z. Ben Slimene, M. Ben Mbarek, N. Faouel, A. Ben Haouala, F. Zaafrane, A. Mhalla

**Affiliations:** 1 Psychiatry Research Laboratory LR05ES10 (Vulnerability To Psychoses), Faculty Of Medicine Of Monastir, University Of Monastir; 2 Psychiatry, Fattouma Bourguiba university hospital, Monastir; 3 UPSAT, Sousse, Tunisia

## Abstract

**Introduction:**

Alcohol consumption among young adults constitutes a major public health concern, given its association with a range of adverse health, academic, and social outcomes. This population is particularly susceptible to hazardous drinking behaviors due to several factors. A comprehensive understanding of its prevalence and contributing risk factors is essential for informing evidence-based prevention strategies and targeted interventions within this demographic.

**Objectives:**

To assess the prevalence and risk factors associated with alcohol use among nursing students at a private institute in Tunisia.

**Methods:**

A cross-sectional analytical study was conducted involving 300 nursing students at a private higher education institute in Tunisia. Data were collected using a questionnaire, and alcohol dependence was assessed using CAGE-DETA test.

**Results:**

The study included 300 nursing students, predominantly female (63%), with most participants aged 20–21 years (49%). All respondents were single, and the majority (96%) had not repeated an academic year. Over half (54.3%) lived with their families. 33.7% of participants reported having used psychoactive substances, with alcohol being the most common after tabacco (28%). About 57.1% of students showed potentially risky or high alcohol consumption, while 42.9% reported low or no use. The mean consumption score was 1.79, indicating moderate overall use, with a subset at risk of misuse. Several influencing factors were identified. Alcohol use increased significantly with age (r = 0.259, p < 0.001) and was more prevalent among males (20%) than females (8%) (p < 0.001). Although academic level was not significant (p = 0.452), first-year students showed higher consumption (10.7%). Repeating a year showed a marginal association (p = 0.102). Students living away from family reported higher use (16% vs. 12%, p = 0.014). Multivariate analysis shows age and male sex are significant predictors of alcohol consumption. Older students and males are more likely to consume alcohol, while females have a lower likelihood (OR = 0.138). Place of residence shows a non-significant trend toward affecting consumption.

**Conclusions:**

Alcohol consumption is prevalent among college students. These findings underscore the need for targeted prevention strategies addressing high-risk groups, particularly younger male students and those living away from family, to reduce harmful alcohol consumption in university settings.

**Disclosure of Interest:**

None Declared

